# Piezoelectricity Regulating Immune Osteogenesis in Osteoporosis

**DOI:** 10.34133/bmef.0146

**Published:** 2025-07-02

**Authors:** Liyun Wang, Jialiang Zhou, Shengjie Jiang, Xiaoling Deng, Wenbin Zhang, Kaili Lin

**Affiliations:** Department of Oral and Cranio-Maxillofacial Surgery, Shanghai Ninth People’s Hospital, Shanghai Jiao Tong University School of Medicine; College of Stomatology, Shanghai Jiao Tong University; National Center for Stomatology; National Clinical Research Center for Oral Diseases; Shanghai Key Laboratory of Stomatology; Research Unit of Oral and Maxillofacial Regenerative Medicine, Chinese Academy of Medical Sciences, Shanghai 200011, China.

## Abstract

**Objective:** This study aims to investigate the regulatory effects of piezoelectricity on the inflammatory microenvironment in osteoporosis treatment. **Impact Statement:** Recent studies have extensively explored the impact of piezoelectric materials on macrophage polarization and cytokine secretion. However, the effects of piezoelectricity on macrophages for the regulation of immune osteogenesis in osteoporosis remain poorly understood. This study provides novel insights into the regulatory role of piezoelectricity in macrophage modulation within osteoporotic diseases. **Introduction:** The overexpression of various inflammatory factors in osteoporosis exacerbates bone metabolism imbalance. Macrophage polarization plays a pivotal role in inflammation regulation and tissue repair. Therefore, investigating the regulatory effects of piezoelectricity on macrophage polarization is crucial for improving the inflammatory microenvironment and fostering an immune environment conducive to osteoporotic bone regeneration. **Methods:** This study fabricated polarized potassium sodium niobate ceramic (PKNN) piezoelectric bioceramics using solid-phase sintering and high-pressure polarization techniques, and investigated their regulatory effects on macrophage polarization, anti-inflammatory factor expression, and osteogenic differentiation bone marrow mesenchymal stem cells derived from ovariectomized rats (rBMSCs-OVX). **Results:** PKNN substantially promotes M2 macrophage polarization and enhances anti-inflammatory factor expression, effectively suppressing inflammatory responses. Further studies demonstrate that PKNN, by modulating macrophages, indirectly regulates osteoblast gene expression, improving the inhibitory effects of the pathological microenvironment on osteogenic differentiation of rBMSCs-OVX. **Conclusion:** This research provides important theoretical evidence for the development of immunomodulatory osteoporotic bone regeneration materials driven by piezoelectricity.

## Introduction

Osteoporosis (OP) is a systemic bone disorder marked by diminished bone density and degradation of bone structure. Its pathophysiological mechanism involves the disruption of the dynamic balance between bone resorption and bone formation, resulting in increased bone fragility and elevated fracture risk [1]. This disease is particularly prevalent in the elderly population; a study revealed that approximately 8.9 million people worldwide experience fractures annually [2]. The annual healthcare costs associated with fractures related to postmenopausal OP in the United States amount to $57 billion and are projected to exceed $95 billion by 2040 [3]. Studies have shown that the bone regeneration capacity of OP patients is impaired, manifested by the reduced mechanical properties of bone callus, such as decreased compressive strength, peak failure load, and bending stiffness. Additionally, the structure of trabecular bone is often compromised, characterized by reduced connectivity and fewer trabeculae [4].

In postmenopausal women, estrogen deficiency triggers an imbalance in bone remodeling, leading to bone loss [5]. Postmenopausal women exhibit marked alterations in the quantity and function of immune cells, which may promote the development of OP by modulating the bone immune microenvironment [6]. The decline in estrogen levels combined with aging induces chronic low-grade inflammation in the body, which disrupts bone metabolic balance by activating signaling pathways such as nuclear factor κB (NF-κB) and promoting pro-inflammatory cytokines [e.g., tumor necrosis factor-α (TNF-α), interleukin-1 (IL-1), and IL-6], thereby further accelerating the progression of OP [7]. Macrophages, as a crucial component of the innate immune system, originate from the monocyte lineage and are widely distributed throughout various tissues. They primarily participate in pathogen clearance, removal of cellular debris, and regulation of inflammatory responses [8].

Macrophages exhibit high plasticity, enabling them to dynamically switch between pro-inflammatory (M1) and anti-inflammatory/pro-regenerative (M2) phenotypes. In postmenopausal women, the number of monocytes in bone marrow notably increases, accompanied by macrophage activation and imbalance in M1/M2 polarization. This imbalance exacerbates oxidative stress, leading to elevated levels of reactive oxygen species (ROS), which further amplify the inflammatory response [9]. The interaction between ROS and inflammation disrupts the dynamic balance of bone metabolism by inhibiting osteoblast differentiation, promoting osteoclast activation, inducing osteocyte apoptosis, and increasing the RANKL/OPG ratio, ultimately leading to the development of OP [10,11].

The clinical repair of osteoporotic bone fracture faces unique challenges: Reduced bone density and the destruction of trabecular microstructure lead to insufficient bone–implant interface strength, resulting in a loosening rate of traditional internal fixation systems (such as plates and screws) that can be 3 to 5 times higher than that of healthy bone [12]. Autologous bone grafting, with its inherent osteoconductivity, osteoinductivity, and lack of immune rejection, is regarded as the “gold standard” for bone defect repair. However, its clinical application is limited by donor site complications, the trauma of secondary surgeries, and insufficient bone supply. Although allogeneic and xenogeneic bone grafting can overcome the limitations of autologous bone sources, their clinical use is still constrained by biological issues such as high disease transmission risks and severe immune rejection [13]. Therefore, new repair biomaterials need to be developed.

Bone tissue exhibits a endogenous electric field, attributed to the piezoelectric effect generated by its collagen matrix [14]. This mechanism converts physiological compressive loads into piezoelectric stimuli, not only maintaining the dynamic balance of bone metabolism but also providing a theoretical foundation for the biomimetic design of bone repair materials [15,16]. Biomaterials based on piezoelectric principles can generate low-intensity bioelectric currents within tissues, simulating the endogenous electric field, thereby regulating key biological processes such as intercellular communication and ion transport, markedly promoting tissue regeneration [17,18]. Their sensitivity to minor physical stresses (e.g., cellular traction forces) and noninvasive driving capabilities [e.g., vibration or ultrasound (US)] make them a sustainable self-powered therapeutic strategy in bone tissue regeneration engineering [19].

Recent studies have shown that piezoelectric catalytic nanoparticles induce intracellular ROS generation through US, activating the innate immune response of macrophages and enhancing their phagocytic and bactericidal functions [20]. Additionally, piezoelectric materials regulate immune responses through electrical signals and the microenvironment, driving macrophage polarization toward the M2 phenotype and stimulating the secretion of anti-inflammatory and pro-healing growth factors [e.g., IL-10 and transforming growth factor-β (TGF-β)] [21]. Given the pivotal role of piezoelectric materials in regulating macrophage polarization and the immune microenvironment, their development and application have become a focal point in current research.

At present, research on the application of piezoelectric materials in osteoporotic bone regeneration remains in the exploratory stage, but recent breakthrough studies have demonstrated their potential. For example, Wu et al. [22] developed a “dual-regulation” bone homeostasis strategy based on a piezoelectric composite film (DAT/KS). Their study confirmed that under US stimulation, the DAT/KS membrane simultaneously enables dynamic piezoelectric signal modulation and controlled release of saikosaponin D (SSD), thereby synergistically promoting osteoblasts differentiation while suppressing osteoclast activity. This dual effect improved bone metabolic imbalance in OP models, providing a novel approach for the application of piezoelectric materials in bone regeneration. In addition to piezoelectric composite membranes, piezoelectric ceramics also demonstrate unique bio-regulatory advantages. However, the application of pure piezoelectric bioceramics for osteoporotic bone regeneration has not been reported until now.

Potassium sodium niobate ceramic (KNN) is a substantial piezoelectric ceramic material [23,24]. In the research of novel piezoelectric materials, the surface potential of polarized KNN (PKNN) ceramics plays a crucial role in promoting biological processes such as protein adsorption and cell proliferation [25]. Recent studies indicate that in the poly(lactic-co-glycolic acid)/zinc-KNN (PLGA/Zn-KNN) scaffold combined with US treatment group, US-mediated electrical stimulation can promote macrophage activation toward the M1 phenotype and enhance the secretion of pro-inflammatory factors, an effect potentially related to its ability to augment the phagocytic function of macrophages against bacteria. Additionally, preliminary studies have demonstrated that this material can promote osteogenic differentiation [26]. However, the role of piezoelectricity on the regulation of osteoporotic immune microenvironment remains unclear.

**Fig. 1.**
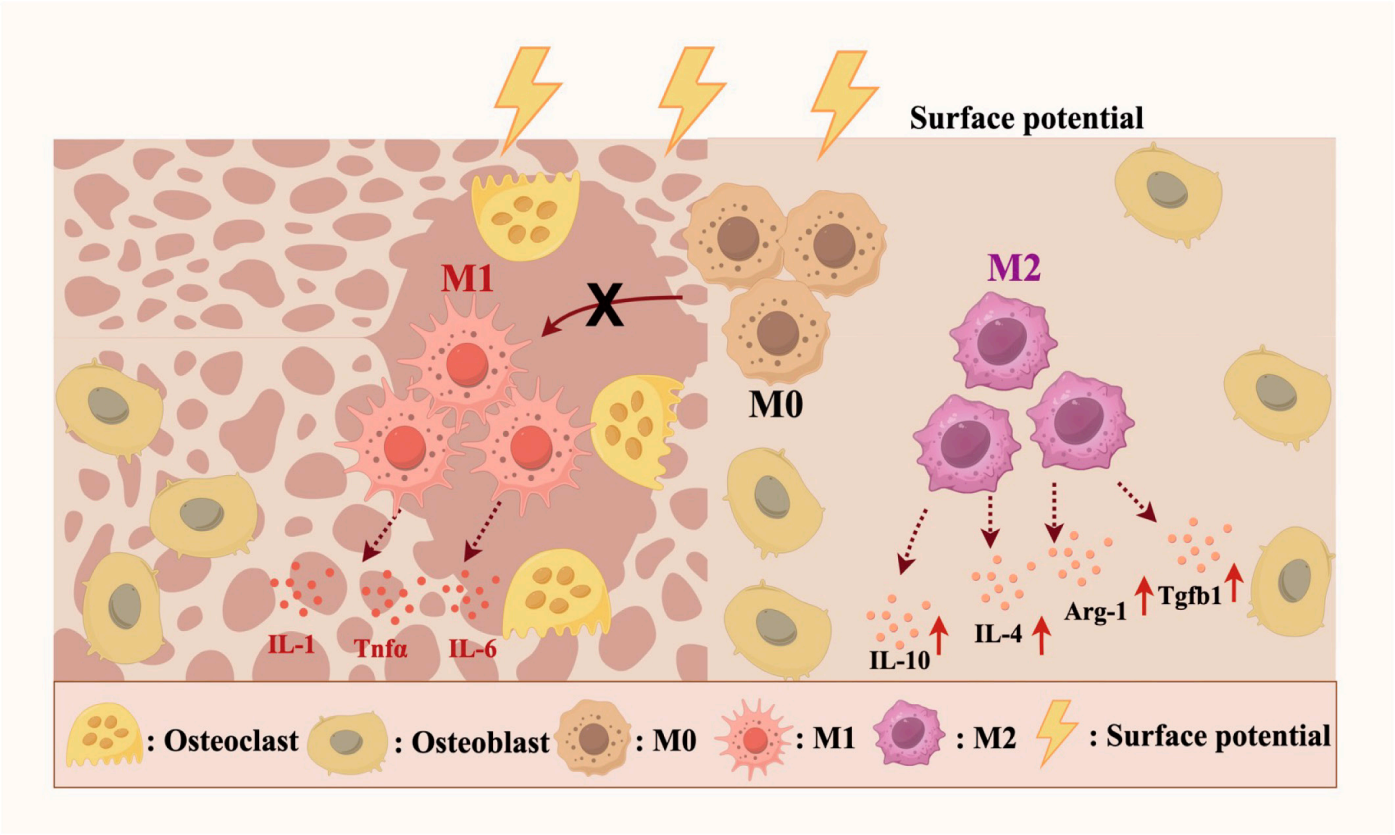
Schematic illustration of the biological functions of PKNN piezoelectric bioceramics in suppressing inflammation based on osteoimmunomodulation (created by Figdraw).

Considering the potential of electrical stimulation in promoting osteogenic differentiation and immunomodulation, this study aims to explore the immunomodulatory role of PKNN in osteoporotic bone repair, providing a theoretical foundation for its potential clinical applications. Specifically, the piezoelectric properties of PKNN may suppress the overactivation of M1 macrophages while promoting their polarization toward the M2 phenotype, thereby reducing the release of pro-inflammatory factors, and increasing the secretion of anti-inflammatory and pro-healing factors. The amelioration of the immune microenvironment not only helps alleviate inflammatory responses in OP but also may accelerate bone tissue regeneration by enhancing osteogenic differentiation (Fig. 1).

## Results and Discussion

### Characterization of KNN/PKNN bioceramics

**Fig. 2.**
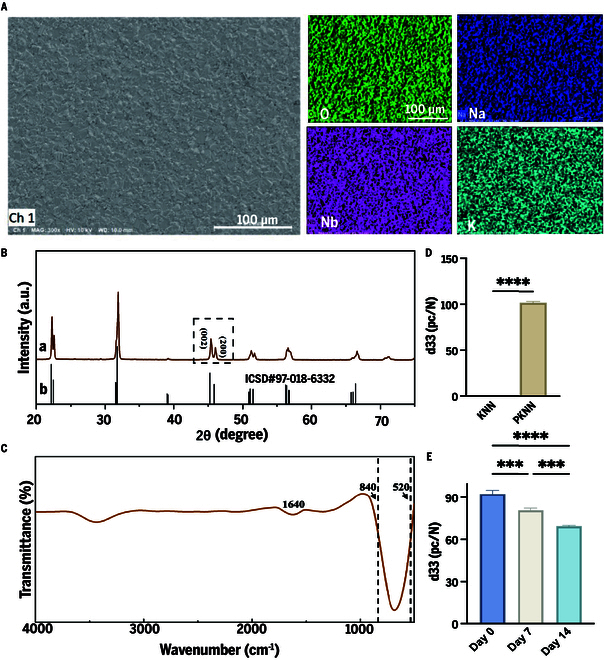
Structural characterization of KNN. (A) SEM and EDS images of KNN bioceramics. (B) XRD diffraction patterns of KNN bioceramics (a) compared with the standard reference pattern (ICSD# 97-018-6332; b). (C) FTIR spectrum of KNN bioceramics. (D) Piezoelectric coefficient d33 of KNN and PKNN bioceramics. (E) Changes in piezoelectric performance (d33) after immersing the material in PBS for 7 and 14 d.

In this study, KNN ceramics were successfully fabricated via solid-phase sintering technology, and their phase composition, structural characteristics, and piezoelectric properties were systematically characterized. Scanning electron microscopy (SEM) reveals that the surface of KNN ceramics exhibits a dense and uniform microstructure, with no apparent pores or cracks observed. This surface characteristic is beneficial for enhancing the mechanical strength and stability of the piezoelectric properties of the ceramics. Additionally, energy-dispersive spectrometer (EDS) analysis confirms that KNN ceramics are composed of 4 elements: Nb, K, Na, and O (Fig. 2A).

X-ray diffraction (XRD) technology was employed to analyze the phase composition of the fabricated ceramic sample. As shown in Fig. 2B, the diffraction peaks in the XRD pattern indicate that the fabricated KNN piezoelectric bioceramics exhibit typical ABO_3_ perovskite-type structural features. Near the 45° diffraction angle, 2 characteristic diffraction double peaks, namely, (002) and (200), were observed. Previous studies in related fields have confirmed that these 2 specific diffraction double peaks play a crucial role in determining the phase structure of KNN piezoelectric ceramics [27]. In the the fabricated KNN ceramics, this ratio of (002) and (200) was approximately 2:1, indicating that the fabricated KNN ceramics exhibited an orthorhombic phase structure.

The Fourier transform infrared (FTIR) spectrum of the KNN bioceramics (Fig. 2C) exhibited absorption bands ranging from 520 cm^−1^ to 840 cm^−1^, which correspond to the characteristic coupling bands of [NbO₆]^7−^ octahedra formed by Nb–O bonds [28]. Based on previous research findings from our group [26], the final optimized corona poling parameters were determined as follows: poling electric field strength of 10 kV/cm, poling time of 40 min, and poling temperature of 225 °C. After the polarization treatment, the d33 coefficient of PKNN reached 92 pC/N, significantly higher than that of KNN, indicating that the polarization process effectively enhances its piezoelectric performance (Fig. 2D). To investigate the stability of PKNN bioceramics in a solution environment, we randomly selected 3 samples for corona polarization treatment. These samples were then immersed in body fluid environment, and their piezoelectric performance was periodically tested. The experimental results are shown in Fig. 2E. After 14 d of immersion, the d33 value of PKNN decreased from the initial (90 ± 4) pC/N to (69 ± 3) pC/N while maintaining relatively high piezoelectric performance, demonstrating good stability in phosphate-buffered saline (PBS) environment.

### Effects of PKNN on macrophage proliferation, adhesion, and morphology

**Fig. 3.**
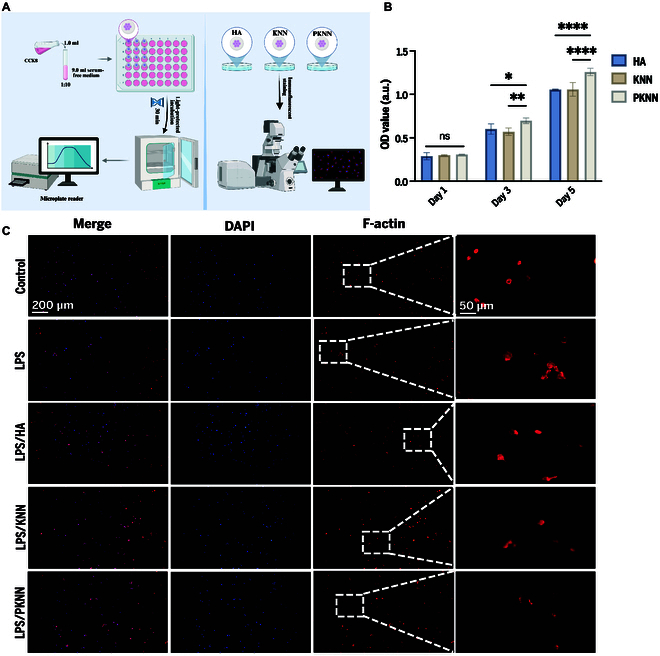
Observation of macrophage proliferation and morphology adhered to different bioceramic surfaces. (A) CCK-8 assay schematic diagram. (B) Effects of HA, KNN, and PKNN on the proliferation of RAW264.7. (C) Immunofluorescence staining images of macrophage morphology in different groups (red: F-actin; blue: DAPI; scale bar: 100 μm). Fluorescence images of magnified macrophage morphology in different experimental groups (scale bar: 50 μm).

The biocompatibility of materials is an indispensable key property for their clinical applications. Hydroxyapatite (HA), as a widely applied bioactive material in clinical settings, has been extensively studied and validated for its physicochemical properties and biocompatibility. Given its recognized reliability and reproducible experimental data, this study selected HA as the control material in vitro experiments [29]. To evaluate the biocompatibility of the fabrucated materials, RAW264.7 cells were seeded onto the surfaces of HA, KNN, and PKNN. Cell proliferation was evaluated using the cell counting kit-8 (CCK-8) assay after 1, 3, and 5 d of culture (Fig. 3A). On day 3, the optical density (OD) value of the PKNN group was significantly greater than that of the KNN group, suggesting that the surface potential promotes cell proliferation. By day 5, the OD value of the PKNN group further increased, surpassing that of the HA group significantly (Fig. 3B). These results demonstrate that the polarized PKNN material significantly enhances RAW264.7 proliferation. This difference may be attributed to the unique surface potential of PKNN.

Lipopolysaccharide (LPS) can induce macrophages to secrete pro-inflammatory factors (such as TNF-α and IL-6) and promote their polarization toward the M1 phenotype [30]. Before the formal experiment, RAW264.7 cells were exposed to LPS for 24 h to simulate the inflammatory cell state in the OP microenvironment. Fluorescence imaging results validated the influence of surface potential on the morphological characteristics of different macrophage phenotypes. As shown in Fig. 3C, macrophages stimulated with LPS exhibited irregular, larger cell shapes, consistent with the classic pro-inflammatory M1 phenotype [31,32]. Macrophages on HA and KNN also predominantly displayed irregular shapes, suggesting that these materials may not alter the pro-inflammatory phenotype of macrophages. In contrast, macrophages on PKNN were mostly spindle-shaped or round, morphological characteristics highly consistent with the anti-inflammatory M2 phenotype. M2 macrophages are typically involved in tissue repair and immune regulation, and their morphological changes (such as spindle or round shapes) are closely related to their functions. These results confirm that PKNN may promote macrophage polarization toward the M2 phenotype by regulating their morphology and function.

### PKNN regulates the M1–M2 polarization of macrophages

**Fig. 4.**
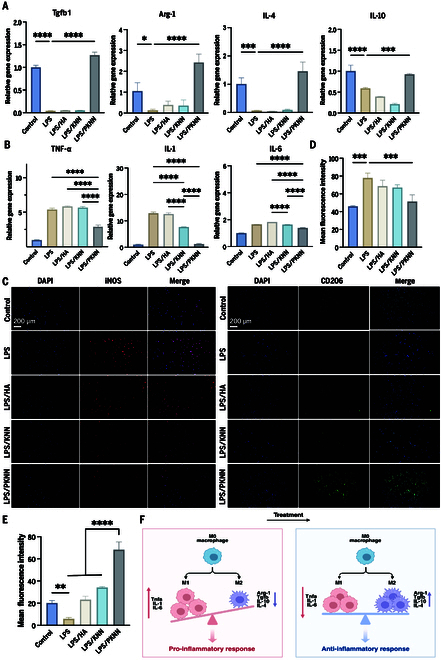
PKNN bioceramics suppress inflammatory response and promote macrophage polarization toward anti-inflammatory phenotype. (A) Expression levels of anti-inflammatory marker genes (*IL-4*, *IL-10*, *Arg-1*, and *Tgfb1*) after coculturing with different material groups. (B) Expression levels of proinflammatory marker genes (*IL-6*, *IL-1*, and *TNF-α*) in RAW264.7 after coculturing with different material groups. (C) Immunofluorescence staining images of M1 and M2 phenotype marker of different material groups (red: iNOS; green: CD206; blue: DAPI; scale bar: 200 μm). (D) Quantitative statistics of M1 phenotype marker of different material groups. (E) Quantitative statistics of M2 phenotype marker of different material groups. (F) Schematic diagram of the anti-inflammatory effect of surface potential (created with BioRender.com).

To further explore the regulatory role of PKNN bioceramics on the inflammatory microenvironment, we evaluated the effect of PKNN ceramics on macrophage polarization. Quantitative reverse transcription polymerase chain reaction (qRT-PCR) results showed that the PKNN group significantly enhanced the expression of anti-inflammatory factors associated with M2 macrophages (Arg-1, IL-4, IL-10, and Tgfb1) (Fig. 4A). Numerous studies have demonstrated that M2 macrophages effectively suppress inflammatory responses by secreting high levels of anti-inflammatory factors, creating favorable conditions for tissue repair [33]. Therefore, the advantages of PKNN in promoting M2 macrophage polarization and up-regulating anti-inflammatory factor expression suggest its superior ability to modulate the inflammatory microenvironment, which provides essential conditions for the repair and reconstruction of damaged bone tissue.

M1 macrophages amplify inflammatory responses and exacerbate tissue damage by secreting pro-inflammatory factors and ROS. In the PKNN group, the expression levels of TNF-α, IL-1, and IL-6 were significantly reduced compared to the control group (Fig. 4B), indicating that the surface potential of PKNN piezoelectric bioceramics can effectively suppress the activation of M1 macrophages, thereby alleviating inflammatory responses. The expression of M1 marker [inducible nitric oxide synthase (iNOS)] and M2 marker (CD206) in RAW264.7 was assessed using immunofluorescence staining. As illustrated in Fig. 4C, within the inflammatory microenvironment (LPS treatment group), the number of iNOS^+^ cells increased significantly, while the number of CD206^+^ cells decreased markedly, demonstrating that LPS effectively induced macrophage polarization toward the M1 phenotype. However, PKNN treatment partially reversed this trend, with the LPS/PKNN group exhibiting the most pronounced inhibition of M1 polarization in RAW264.7. This finding was further corroborated by corresponding quantitative analysis (Fig. 4D). Concurrently, the LPS/PKNN group also displayed the most significant enhancement in promoting RAW264.7 polarization toward the M2 phenotype (Fig. 4E). These results indicate that PKNN effectively suppresses the activation of M1 macrophages and promotes the activation of M2 macrophages, suggesting its potential role in the regulation of inflammation.

In summary, the advantages of PKNN in promoting M2 macrophage polarization and up-regulating anti-inflammatory factor expression suggest its superior ability to modulate the inflammatory microenvironment (Fig. 4F). PKNN can more effectively guide macrophages toward an anti-inflammatory phenotype, inhibit inflammatory cascades, and provide essential conditions for the repair and reconstruction of damaged bone tissue. We speculated that the surface potential of PKNN bioceramics may influence ion channels or signaling molecules on the macrophage surface, altering intracellular signal transduction and thereby regulating macrophage polarization.

### PKNN–macrophage conditioned medium promoted osteogenic differentiation of rBMSCs-OVX

During tissue healing, the effective migration of osteoblasts to the injury site is a critical step. However, the inflammatory microenvironment in OP severely impairs cell migration capacity, which may be one of the key reasons for delayed bone repair. As crucial effector cells in immune regulation, macrophages secrete a variety of bioactive factors (such as growth factors, cytokines, and chemokines) that act on the surrounding microenvironment through autocrine and paracrine pathways, thereby regulating the proliferation, differentiation, and migration of mesenchymal stem cells. Inflammatory factors not only inhibit cell migration but also interfere with their proliferation and differentiation functions, thereby hindering the tissue repair process [34]. The above in vitro experiments demonstrated that PKNN can promote macrophage polarization toward the M2 phenotype. Based on this finding, we hypothesized that M2 macrophages induced by PKNN may play a key role in rescuing the functions of cells involved in osteogenic differentiation. To test this hypothesis, we used ovariectomized rBMSCs-OVX and cocultured them with macrophage-conditioned media (control, LPS, and LPS/PKNN). Following this, we assessed the migratory capabilities of rBMSCs-OVX as well as their abilities to undergo osteogenic differentiation.

**Fig. 5.**
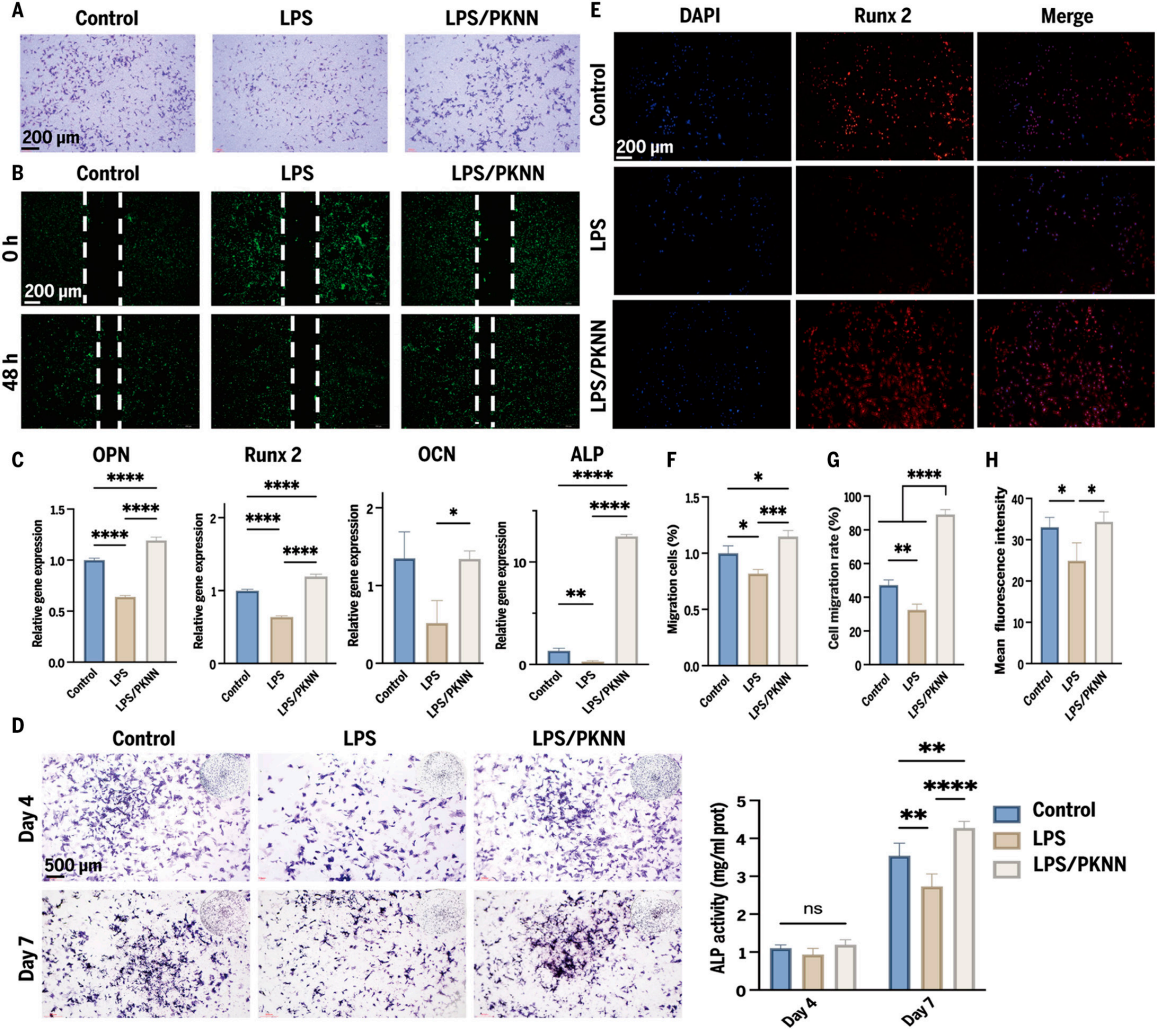
PKNN conditioned medium promotes migration and osteogenic differentiation of rBMSCs-OVX. (A) Transwell experiment (scale bar: 200 μm). (B) Scratch experiment (scale bar: 200 μm). (C) Expression status of osteogenic marker genes in rBMSCs-OVX after coculturing with different conditioned media for 4 d. (D) ALP staining images and semiquantitative analysis of different conditioned media stimulated for 4 and 7 d (scale bar: 500 μm). (E) Immunofluorescence staining images of different conditioned media stimulated for 4 day (red: Runx 2; blue: DAPI; scale bar: 200 μm). (F) Quantitative analysis of cell migration in transwell. (G) Quantitative analysis of cell migration in scratch assay. (H) Quantitative statistics of immunofluorescence staining.

Therefore, to evaluate the rescuing effect of PKNN on the migratory ability of rBMSCs-OVX, we conducted systematic analyses using transwell migration assays (Fig. 5A) and scratch assays (Fig. 5B). The results demonstrated that, compared with the control group, the LPS-treated group significantly suppressed cell migration, whereas the migration ability of the PKNN-treated group was markedly restored. Transwell quantification revealed that the number of migrated cells in the PKNN group was significantly higher than that in the control group (Fig. 5F). The scratch assay further confirmed that the cell migration rate was significantly increased in the PKNN group (Fig. 5G). We speculate that this enhanced migration is primarily attributed to the presence of various growth factors and chemokines in the PKNN-conditioned medium, which not only stimulate cell proliferation and differentiation but also play a crucial guiding role in promoting cell migration.

Additionally, we used qRT-PCR to detect the expression levels of osteogenesis-related genes in conditioned media (Fig. 5C). The results showed that on day 4, the inflammatory environment induced by LPS significantly suppressed the expression of osteogenic genes, a phenomenon possibly related to the interference of inflammatory factors with osteogenic signaling pathways. In contrast, the macrophage-conditioned medium from the PKNN group significantly reversed the inhibitory effects of the inflammatory microenvironment on osteogenic gene expression and promoted the expression of related osteogenic markers compared to other groups. These results demonstrate that PKNN piezoelectric ceramics play a positive role in regulating osteogenic differentiation within the inflammatory microenvironment.

We further evaluated the effects of macrophage-conditioned medium on the osteogenic differentiation ability of rBMSCs-OVX through alkaline phosphatase (ALP) staining and semiquantitative analysis. As shown in Fig. 5D, the ALP expression level was highest in the PKNN-treated macrophage-conditioned medium, significantly exceeding that of the control and LPS groups. This indicates that PKNN can effectively reverse the inhibitory effects of the inflammatory microenvironment on osteogenic differentiation and significantly enhance the osteogenic potential of rBMSCs-OVX. Based on previous experiments, PKNN material can induce macrophage polarization toward the M2 phenotype, and cytokines secreted by M2 macrophages (such as IL-10 and IL-4) can suppress inflammatory responses, creating favorable conditions for osteogenic differentiation. Additionally, M2 macrophages secrete various bioactive molecules that promote the osteogenic differentiation of mesenchymal stem cells, accelerating bone tissue repair and regeneration [35]. Therefore, we propose that PKNN can indirectly influence the gene expression and functional activity of osteoblasts by regulating macrophage polarization states. Runx 2 plays a crucial role as a transcription factor in the differentiation of osteoblasts, as it influences the expression of early osteogenic genes, including ALP, and facilitates the transition of osteoprogenitor cells into pre-osteoblasts [36].

Runx 2 serves as a vital transcription factor involved in the processes of osteoblast differentiation and bone formation, playing a crucial role in the proliferation, maturation, and differentiation of osteoblasts. Its expression level directly reflects the osteogenic potential of cells [37]. As shown in Fig. 5E and H, in the control group, rBMSCs-OVX exhibited moderate Runx 2-positive signals, indicating a certain baseline of osteogenic differentiation. However, in the LPS-treated group, the expression of Runx 2 was significantly reduced, suggesting that the inflammatory microenvironment inhibited the osteogenic differentiation ability of rBMSCs-OVX. In other words, inflammatory factors interfere with osteoblast differentiation and function by suppressing Runx 2 expression. Notably, in the LPS/PKNN-treated group, the expression of Runx 2 was significantly restored and even exceeded the level of the control group. This indicates that PKNN can effectively reverse the inhibitory effects of the inflammatory microenvironment on Runx 2 expression, likely by promoting macrophage polarization toward the M2 phenotype and creating a microenvironment conducive to osteogenic differentiation.

## Conclusion

The effect of piezoelectricity on the osteogenesis of OP is still unclear. Based on piezoelectric PKNN bioceramics, we demonstrated that piezoelectricity has substantial advantages in promoting M2 macrophage polarization and up-regulating the expression of anti-inflammatory factors, indicating its ability to modulate the inflammatory microenvironment and effectively guide macrophages toward an anti-inflammatory phenotype. Further research revealed that piezoelectricity indirectly influences the gene expression and functional activity of osteoblasts by regulating macrophage polarization states, reversing the inhibitory effects of the inflammatory microenvironment on osteogenic gene expression and promoting the expression of related osteogenic markers of rBMSCs-OVX. These results highlight the crucial role of piezoelectricity in regulating the immune microenvironment and osteogenic differentiation, providing an important theoretical foundation for developing immunomodulation-based bone repair materials for OP.

## Materials and Methods

### Materials

The following materials were used: high-purity Na_2_CO_3_ (99.8%, analytically pure, Sinopharm Chemical Reagent Co., China), K_2_CO_3_ (99.8%, analytically pure, Sinopharm Chemical Reagent Co., China), SrCO_3_ (99.9%, analytically pure, Sinopharm Chemical Reagent Co., China), and Nb_2_O_5_ (99.99%, analytically pure, Sinopharm Chemical Reagent Co., China), and HA (Aladdin, biomedical grade).

### Fabrication of HA, KNN, and PKNN bioceramics

Synthesis of KNN: K_2_CO_3_, Na_2_CO_3_, Nb_2_O_5_, and anhydrous ethanol were ball-milled for 10 h, dried, and sieved through a 200-μm mesh. The mixture was then pressed into 25-mm-diameter discs and pre-sintered at 850 °C for 4 h. After crushing and sieving through a 150-μm mesh, 8 wt % polyvinyl alcohol (PVA, Aladdin) was added for granulation. The granules were pressed into 10-mm-diameter ceramic discs under 10-MPa pressure, sintered at 600 °C for 3 h, and subsequently sintered at 1,050 °C for 2 h. PKNN was obtained by polarizing the KNN ceramics with corona discharge [26]. HA was fabricated using the same method and sintered at 1,100 °C for 3 h as a control group.

### Characterization of the HA, KNN, and PKNN bioceramics

The characteristic absorption peaks of KNN were analyzed using FTIR. The crystal structure of KNN was characterized by XRD with a scanning range of 20° to 70° (2θ). The piezoelectric coefficient d33 was measured using a piezoelectric d33 meter (YE2730A, China Electronics Corporation).

### Cell culture

#### RAW264.7 culture

RAW264.7 cells (CRL-1730, ATCC, USA) were seeded in culture dishes containing Dulbecco’s modified Eagle’s medium supplemented with 10% fetal bovine serum (FBS) (Gibco, USA) and 1% antibiotic–antimycotic solution (Gibco, USA). The cells were then cultured in an environment maintained at 37 °C with 5% CO_2_.

#### Extraction and culture of rBMSCs-OVX

The OVX model rats were euthanized by cervical dislocation, and the femurs and tibias were collected. The ends of the bones were removed to expose the marrow cavity. The marrow cavity was flushed with α-MEM (minimum essential medium) (Gibco, USA) containing 15% FBS and 1% penicillin–streptomycin until the marrow appeared translucent and white. The cell suspension was then seeded into culture dishes and incubated at 37 °C with 5% CO_2_. When the cells reached approximately 90% confluence, they were passaged for subsequent experiments.

### Cell proliferation and adhesion assay

#### Cell proliferation assay

RAW264.7 cells were seeded at a density of 3 × 10^3^ cells per well onto the surfaces of HA, KNN, and PKNN ceramics, and the culture medium was replaced every other day. On days 1, 3, and 5, the old medium was aspirated, and the working solution was added, followed by incubation in a 37 °C incubator protected from light for 40 min. Subsequently, 100 μl of supernatant from each well was transferred to a 96-well plate, and the absorbance at 450 nm was measured using a microplate reader.

#### Cell adhesion assay

RAW264.7 cells were seeded at a density of 1 × 10^4^ cells per well in 48-well plates and onto the surfaces of material groups. The cells were stimulated with LPS for 24 h, and the cells were incubated for 24 h. The cells were then fixed using 4% paraformaldehyde, washed with PBS, and permeabilized. Actin-Tracker was added, and the cells were incubated at room temperature in the absence of light. After washing by PBS, 4′,6-diamidino-2-phenylindole (DAPI) was added, and the cells were incubated for 5 min in the dark. Following another PBS wash, the cells were examined and imaged under a microscope.

### qRT-PCR analysis of inflammation-related genes

RAW264.7 cells were separately seeded into 48-well plates and onto the surfaces of 3 material groups. The cells were stimulated by LPS for 24 h, followed by replacement with complete medium and incubation for 72 h. RNA was extracted and converted into cDNA through reverse transcription, and qPCR was performed using primer sequences listed in Table S1.

### M1 immunofluorescence staining

In parallel, RAW264.7 cells were seeded into 48-well plates and onto the surfaces of the 3 material groups, stimulated with LPS for 24 h, and then incubated with complete medium for 72 h. Immunofluorescence staining experiments were conducted to assess M1 polarization of macrophages. Ultimately, the samples were examined and imaged using a microscope, and fluorescence intensity was quantified using ImageJ software.

### M2 immunofluorescence staining

RAW264.7 cells were seeded onto the surfaces of 3 different materials placed in a 48-well plate. After stimulation with LPS for 24 h, the medium was replaced with complete medium, and the cells were further incubated for 72 h. For fluorescence staining of the M2 macrophage marker CD206, an anti-CD206 antibody (1:200) was used, followed by incubation at room temperature in the dark for 1 h. Finally, the samples were observed and photographed under a fluorescence microscope, and the fluorescence intensity was analyzed using ImageJ software.

### PKNN–macrophage conditioned medium preparation

RAW264.7 cells were plated at a density of 1 × 10^5^ cells per well onto ceramic surfaces, stimulated by LPS for 24 h, and then incubated with complete medium for 72 h. The supernatants were collected, centrifuged, filtered, and stored at −80 °C. The conditioned media from these groups were labeled as control, LPS, and LPS/PKNN. For subsequent experiments, the conditioned media were mixed with complete medium at a ratio of 1:2.

### Biological effects of PKNN-conditioned medium on rBMSCs-OVX

#### Transwell assay

rBMSCs-OVX were seeded at a density of 2 × 10^4^ cells per well into the Transwell chambers. Serum-free conditioned medium was added to the upper chamber, while complete medium containing 10% serum was added to the lower chamber. The cells were then incubated at 37 °C for 24 h. After incubation, the cells were fixed with 4% paraformaldehyde and washed. Crystal violet staining was performed, followed by washing. The upper chamber was wiped clean, and the migrated cells were examined and photographed under a stereomicroscope.

#### Scratch assay

rBMSCs-OVX were seeded at a density of 1 × 10^5^ cells/well in a 12-well plate, and a scratch assay was performed after the cells reach 100% confluency. The cells were subsequently fixed with paraformaldehyde, washed, and then stained with calcein-AM. After incubation in the dark for 20 min, the staining solution was removed, and the cells were examined and photographed under a fluorescence microscope. After adding conditioned medium and incubating for 24 h, the cells were fixed, stained, and observed under the microscope.

#### qRT-PCR analysis

rBMSCs-OVX were plated at a density of 1 × 10^5^ cells in each well of 6-well plates and cultured for 4 d, with the conditioned medium being replaced every 3 d. RNA was extracted and converted into cDNA through reverse transcription, followed by qPCR analysis using primer sequences provided in Table S2.

#### ALP staining and semiquantitative analysis

rBMSCs-OVX were plated at a density of 1 × 10^4^ cells per well using 48-well plates, within the conditioned medium being changed every 3 d. ALP staining was performed on days 4 and 7, with observations and photography done under a stereomicroscope. Also, after 4 and 7 d, the cells were lysed and centrifuged. The protein concentration was assessed using the bicinchoninic acid (BCA) assay at 562 nm, while the alkaline phosphatase (AKP) assay at 560 nm was utilized to measure ALP activity.

#### Runx 2 immunofluorescence staining

rBMSCs-OVX were introduced at a concentration of 3 × 10^4^ cells per well within 24-well plates and cultured for 4 d, with conditioned medium replaced every 3 d. Immunofluorescence staining was then performed. The cells were then examined and captured using a fluorescence microscope, and the fluorescence intensity was quantified using ImageJ software.

### Statistical analyses

Data analysis was performed using GraphPad Prism 8 statistical software. Quantitative results are expressed as mean ± standard deviation (SD). Each experimental group included at least 3 samples (*n* ≥ 3). Comparisons between 2 groups were conducted using an independent samples *t* test, while comparisons among multiple groups were performed using one-way analysis of variance (ANOVA). For multifactorial comparisons, 2-way ANOVA was utilized. Statistical significance is denoted by asterisks, with * representing *P* < 0.05, ** representing *P* < 0.01, *** representing *P* < 0.001, and **** representing *P* < 0.0001.

## Data Availability

Data will be provided upon request.

## References

[1] Gopinath V. Osteoporosis. Med Clin North Am. 2023;107(2):213–225. 36759092 10.1016/j.mcna.2022.10.013

[2] Wilson DJ. Osteoporosis and sport. Eur J Radiol. 2019;110:169–174. 30599856 10.1016/j.ejrad.2018.11.010

[3] Walker MD, Shane E. Postmenopausal osteoporosis. N Engl J Med. 2023;389(21):1979–1991. 37991856 10.1056/NEJMcp2307353

[4] Zhang X, Yang B, Feng L, Xu X, Wang C, Lee Y-W, Wang M, Lu X, Qin L, Lin S, et al. Augmenting osteoporotic bone regeneration through a hydrogel-based rejuvenating microenvironment. Bioact Mater. 2024;41:440–454. 39188381 10.1016/j.bioactmat.2024.07.036 PMC11347042

[5] Song S, Guo Y, Yang Y, Fu D. Advances in pathogenesis and therapeutic strategies for osteoporosis. Pharmacol Ther. 2022;237: Article 108168. 35283172 10.1016/j.pharmthera.2022.108168

[6] Fischer V, Haffner-Luntzer M. Interaction between bone and immune cells: Implications for postmenopausal osteoporosis. Semin Cell Dev Biol. 2022;123:14–21. 34024716 10.1016/j.semcdb.2021.05.014

[7] Zhang Z, Tao Z, Zhang Z, Zhang W, Zhang X, Xu X, Gao R, Tao X, Zhou X. Single-cell transcriptomic analysis reveals AP-1 downregulation remodels bone marrow environment and contributes to osteopenia in ovariectomized mice. J Orthop Translat. 2025;52:1–13. 40213116 10.1016/j.jot.2025.03.001 PMC11981807

[8] Zhao C, Yang Z, Li Y, Wen Z. Macrophages in tissue repair and regeneration: Insights from zebrafish. Cell Regen. 2024;13(1):12. 38861103 10.1186/s13619-024-00195-w PMC11166613

[9] Clark D, Brazina S, Yang F, Hu D, Hsieh CL, Niemi EC, Miclau T, Nakamura MC, Marcucio R. Age-related changes to macrophages are detrimental to fracture healing in mice. Aging Cell. 2020;19(3): Article e13112. 32096907 10.1111/acel.13112 PMC7059136

[10] Altindag O, Erel O, Soran N, Celik H, Selek S. Total oxidative/anti-oxidative status and relation to bone mineral density in osteoporosis. Rheumatol Int. 2008;28(4):317–321. 17823800 10.1007/s00296-007-0452-0

[11] Fatokun AA, Stone TW, Smith RA. Responses of differentiated MC3T3-E1 osteoblast-like cells to reactive oxygen species. Eur J Pharmacol. 2008;587(1–3):35–41. 18448093 10.1016/j.ejphar.2008.03.024

[12] Das C, Das PP. Role of augmentation in the fixation of osteoporotic fractures. Indian J Orthop. 2025;59(3):294–299. 40201925 10.1007/s43465-024-01323-z PMC11973026

[13] Brochu BM, Sturm SR, Goncalves JAKDQ, Mirsky NA, Sandino AI, Panthaki KZ, Panthaki KZ, Nayak VV, Daunert S, Witek L, et al. Advances in bioceramics for bone regeneration: A narrative review. Biomimetics. 2024;9(11):690. 39590262 10.3390/biomimetics9110690 PMC11592113

[14] Cui J, Yu B, Li D, Fu Z, Yang X, Jiang L, Wang X, Lin K. Remodeling electrophysiological microenvironment for promoting bone defect repair via electret hybrid electrospun fibrous mat. Adv Fiber Mater. 2024;6:1855–1873.

[15] Vasquez-Sancho F, Abdollahi A, Damjanovic D, Catalan G. Flexoelectricity in bones. Adv Mater. 2018;30(9):1705316. 10.1002/adma.201705316 29345377

[16] Bur AJ. Measurements of dynamic piezoelectric properties of bone as a function of temperature and humidity. J Biomech. 1976;9(8):495–507. 956193 10.1016/0021-9290(76)90066-x

[17] Deng X, Fu Z, Jiang S, Chen X, Cui J, Zhang J, Yang S, Liang Y, Jiang W, Li D, et al. Synergistic effect of electrophysiological microenvironment and bioactive ions for enhancing bone regeneration. Nano Energy. 2024;130: Article 110113.

[18] Yu B, Qiao Z, Cui J, Lian M, Han Y, Zhang X, Wang W, Yu X, Yu H, Wang X, et al. A host-coupling bio-nanogenerator for electrically stimulated osteogenesis. Biomaterials. 2021;276: Article 120997. 34229243 10.1016/j.biomaterials.2021.120997

[19] Nain A, Chakraborty S, Barman SR, Gavit P, Indrakumar S, Agrawal A, Lin Z-H, Chatterjee K. Progress in the development of piezoelectric biomaterials for tissue remodeling. Biomaterials. 2024;307: Article 122528. 38522326 10.1016/j.biomaterials.2024.122528

[20] Liu X, Xu W, Feng J, Wang Y, Li K, Chen Y, Wang W, Zhao W, Ge S, Li J. Adoptive cell transfer of piezo-activated macrophage rescues immunosuppressed rodents from life-threating bacterial infections. Nat Commun. 2025;16(1):1363. 39905015 10.1038/s41467-025-56460-2 PMC11794888

[21] Wu P, Shen L, Liu HF, Zou XH, Zhao J, Huang Y, Zhu YF, Li ZY, Xu C, Luo LH, et al. The marriage of immunomodulatory, angiogenic, and osteogenic capabilities in a piezoelectric hydrogel tissue engineering scaffold for military medicine. Mil Med Res. 2023;10(1):35. 37525300 10.1186/s40779-023-00469-5 PMC10388535

[22] Wu X, Wang T, Zhao J, Zhang L, Liu Z, Chen Y, Luo Y, Liu Y, Chen Y, Jiang H, et al. Ultrasound-responsive piezoelectric membrane promotes osteoporotic bone regeneration via the “two-way regulation” bone homeostasis strategy. Adv Sci. 2025; Article e2504293. 10.1002/advs.202504293 PMC12279238 40289898

[23] Yao T, Chen J, Wang Z, Zhai J, Li Y, Xing J, Hu S, Tan G, Qi S, Chang Y, et al. The antibacterial effect of potassium-sodium niobate ceramics based on controlling piezoelectric properties. Colloids Surf B Biointerfaces. 2019;175:463–468. 30572154 10.1016/j.colsurfb.2018.12.022

[24] Chen Z, Xu K, Zhang X, Wei X. Current status and prospects of additive manufacturing of flexible piezoelectric materials. J Inorg Mater. 2024;39:965.

[25] Chen W, Yu Z, Pang J, Yu P, Tan G, Ning C. Fabrication of biocompatible potassium sodium niobate piezoelectric ceramic as an electroactive implant. Materials. 2017;10(4):345. 28772704 10.3390/ma10040345 PMC5506920

[26] Xu H, Zhuang Y, Fu Z, Cui J, Jiang S, Zhao B, Lin K. Promoted osteogenesis by corona discharge poling induced in electroactive piezoelectric bioceramics. Ceram Int. 2024;50:672–683.

[27] Zheng T, Yu Y, Lei H, Li F, Zhang S, Zhu J, Wu J. Compositionally graded KNN-based multilayer composite with excellent piezoelectric temperature stability. Adv Mater. 2022;34(8):2109175. 10.1002/adma.202109175 34907605

[28] Zhang D, Cheng Z, Cheng J, Shi F, Yang X, Zheng G, Cao M. Hydrothermal preparation and characterization of sheet-like (K*_x_*Na_1-*x*_)NbO*_3_* perovskites. Ceram Int. 2016;42(7):9073–9078.

[29] Alizadeh-Osgouei M, Li Y, Wen C. A comprehensive review of biodegradable synthetic polymer-ceramic composites and their manufacture for biomedical applications. Bioact Mater. 2019;4(1):22–36. 30533554 10.1016/j.bioactmat.2018.11.003 PMC6258879

[30] Xu C, Sarver DC, Lei X, Sahagun A, Zhong J, Na CH, Rudich A, Wong GW. CTRP6 promotes the macrophage inflammatory response, and its deficiency attenuates LPS-induced inflammation. J Biol Chem. 2024;300(1): Article 105566. 38103643 10.1016/j.jbc.2023.105566 PMC10789631

[31] Rios de la Rosa JM, Tirella A, Gennari A, Stratford IJ, Tirelli N. The CD44-mediated uptake of hyaluronic acid-based carriers in macrophages. Adv Healthc Mater. 2017;6(4):1601012. 10.1002/adhm.201601012 27990775

[32] Sun Y, Liu T, Hu H, Xiong Z, Zhang K, He X, Liu W, Lei P, Hu Y. Differential effect of tantalum nanoparticles versus tantalum micron particles on immune regulation. Mater Today Bio. 2022;16: Article 100340. 10.1016/j.mtbio.2022.100340 PMC9278074 35847379

[33] Martin KE, Garcia AJ. Macrophage phenotypes in tissue repair and the foreign body response: Implications for biomaterial-based regenerative medicine strategies. Acta Biomater. 2021;133:4–16. 33775905 10.1016/j.actbio.2021.03.038 PMC8464623

[34] Pajarinen J, Lin T, Gibon E, Kohno Y, Maruyama M, Nathan K, Lu L, Yao Z, Goodman SB. Mesenchymal stem cell-macrophage crosstalk and bone healing. Biomaterials. 2019;196:80–89. 29329642 10.1016/j.biomaterials.2017.12.025 PMC6028312

[35] Xu S, Zhang Y, Dai B, Rao J, Deng F, Zhang SA, Shao H, Li X, Jin Z, Liang T, et al. Green-prepared magnesium silicate sprays enhance the repair of burn-skin wound and appendages regeneration in rats and minipigs. Adv Funct Mater. 2024;34(9):2307439.

[36] Hojo H, Saito T, He X, Guo Q, Onodera S, Azuma T, Koebis M, Nakao K, Aiba A, Seki M, et al. Runx2 regulates chromatin accessibility to direct the osteoblast program at neonatal stages. Cell Rep. 2022;40(10): Article 111315. 36070691 10.1016/j.celrep.2022.111315 PMC9510047

[37] Liu J, Zhang Y, Wu Y, Li G, Ji N, Han R, Tang H, Liu X, Liu H, Wang C, et al. Delivery of m7G methylated Runx2 mRNA by bone-targeted lipid nanoparticle promotes osteoblastic bone formation in senile osteoporosis. Nano Today. 2024;54: Article 102074.

