# Supplementary material for: Piezoelectricity Regulating Immune Osteogenesis in Osteoporosis
Source: BME Front. 2025 Jul 2;6:0146. doi: 10.34133/bmef.0146 (PMC12214298; doi:10.34133/bmef.0146)
Supplement: Supplementary 1 — Tables S1 and S2 [file bmef.0146.f1.docx]

**Supporting information**

**Piezoelectricity Regulating Immune Osteogenesis in Osteoporosis**

Liyun Wang^a, #^, Jialiang Zhou^a, #^, Shengjie Jiang^a, #^, Xiaoling Deng^a^, Wenbin Zhang^a,*^, Kaili Lin^a, *^

^a^Department of Oral and Cranio-Maxillofacial Surgery, Shanghai Ninth People’s Hospital, Shanghai Jiao Tong University School of Medicine; College of Stomatology, Shanghai Jiao Tong University; National Center for Stomatology; National Clinical Research Center for Oral Diseases; Shanghai Key Laboratory of Stomatology; Research Unit of Oral and Maxillofacial Regenerative Medicine, Chinese Academy of Medical Sciences, Shanghai 200011, China.

^#^These authors contributed equally.

***Corresponding author:** lklecnu@aliyun.com & linkaili@sjtu.edu.cn (K. Lin); zwb96493@hotmail.com (W. Zhang).

Table S1. The primer sequences for anti-inflammatory/ pro-inflammatory are shown in the table below.

| Gene | Primer Sequences (F, forward; R, reverse; 5’to 3’) |
| --- | --- |
| *m-β-actin* | F: GGTGGGAATGGGTCAGAAGG  R: GTTGGCCTTAGGGTTCAGGG |
| *m-IL-1* | F: CTGGTACATCAGCACCTCAC  R: AGAAACAGTCCAGCCCATAC |
| *m-IL-6* | F: TGCCTTCTTGGGACTGAT  R: TTGCCATTGCACAACTCTTT |
| *m-Arg-1* | F: ACAAACAAATGTGAATGCAGACCA  R: GAGGCTCCAGGGCATTAGAC |
| *m-Tgfb1* | F: CGTCACTGGAGTTGTACGGC  R: GTTTGGGGCTGATCCCGTTGA |
| *m-IL-4* | F: GGTCTCAACCCCCAGCTAGT  R: GCCGATGATCTCTCTCAAGTGAT |
| *m-IL-10* | F: TGAATTCCCTGGGTGAGAAGC  R: AGACACCTTGGTCTTGGAGCTTATT |
| *m-TNF-a* | F: 5’- AGTGACAAGCCTGTAGCCC -3’  R: 5’- GAGGTTGACTTTCTCCTGGTAT -3’ |

Table S2. The primer sequences for osteogenesis are provided in the table below.

| Gene | Primer Sequences (F, forward; R, reverse; 5’to 3’) |
| --- | --- |
| *β-actin* | F: GTAAAGACCTCTATGCCAACA  R: GTGGATGTCCTTTACCGTCGT |
| *Opn* | F: CCAAGCGTGGAAACACACAGCC  R: GGCTTTGGAACTCGCCTGACTG |
| *Ocn* | F: GGCTTTGGAACTCGCCTGACTG  R: TCACCACCTTACTGCCCTCCTG |
| *Runx 2* | F: ATCCAGCCACCTTCACTTACAAA  R: GGGACCATTGGGAACTGATAGG |
